# LexCHI: A quick lexical test for estimating language proficiency in Chinese

**DOI:** 10.3758/s13428-023-02151-z

**Published:** 2023-07-05

**Authors:** Yun Wen, Yicheng Qiu, Christine Xiang Ru Leong, Walter J. B. van Heuven

**Affiliations:** 1 Department of Applied Linguistics, Xi’an Jiaotong-Liverpool University, Suzhou, China; 2 Laboratoire de Psychologie Cognitive, Aix-Marseille University and Centre National de la Recherche Scientifique, Marseille, France; 3 School of Psychology, University of Nottingham, University Park, Nottingham, NG7 2RD UK; 4 School of Psychology, University of Nottingham Malaysia, Semenyih, Malaysia

**Keywords:** Chinese vocabulary test, Language proficiency, Bilingualism

## Abstract

A prominent methodological issue in cognitive research on bilingualism is the lack of consistency in measuring second language (L2) proficiency. To reduce the inconsistency in L2 proficiency measurements, brief and valid vocabulary tests have been developed as an objective measure of proficiency in a variety of languages (e.g., English, French, Spanish). Here, we present LexCHI, a valid lexical test to measure Chinese proficiency. This freely available short test consists of 60 two-character items presented in simplified Chinese. Although it only takes a few minutes to complete LexCHI, the LexCHI scores in two studies correlated significantly with L2 participants' performance in a translation task and a cloze test. We believe that LexCHI is a useful tool for researchers who need to objectively measure Chinese proficiency as part of their investigations.

## Introduction

It is now common practice to quantify language proficiency of bilingual participants in experimental settings. As reflected in a review of 186 bilingual studies published between 2005 and 2015, 77% of the studies reported bilinguals' language proficiency using a subjective or/and objective assessment (Surrain & Luk, 2019). The rationale behind this research practice is simple: bilingual populations show substantial variation in second language (L2) proficiency, and L2 proficiency is known to affect the representations and processes engaged in bilingual language processing (for reviews, see van Hell & Tanner, 2012; van Hell & Tokowicz, 2010; van Heuven & Dijkstra, 2010).

Despite the need for quantifying bilinguals' proficiency levels, there is little consensus on how to adequately measure L2 proficiency in experiments with bilinguals (for further discussion, see de Bruin, 2019; Hulstijn, 2012). Lacking such consensus, divergent measures of L2 proficiency have been used. For example, the subjective self-rated proficiency, which was provided in more than half of the studies from the above-mentioned review (Surrain & Luk, 2019), may differ in terms of the scales to rate (e.g., a scale of 1 to 7 vs a scale of 1 to 10), the endpoint labels (e.g., a 7-point scale can label its endpoint as "*perfect*", "*native-like*" or "*high proficiency*"), facets of language ability (e.g., speaking vs reading), and how the rating question is framed (e.g., "*How much reading experience do you have with the English language*" used in Lemhöfer & Broersma, 2012, vs "*Indicate how good you consider yourself in reading English*" used in Wen et al., 2018). Regarding objective assessments, Hulstijn (2012) reported that more than 10 different objective proficiency measurements were used in 63 bilingual studies, including the Test of English as a Foreign Language (TOEFL), International English Language Testing System (IELTS), and Test for English Majors (TEM, used in China). The existing diversity in L2 proficiency measures not only hinders across-study comparisons but also renders difficulty in including proficiency in a meta-analytic review (e.g., Lauro & Schwartz, 2017; Wen & van Heuven, 2017b), thus slowing progress in bilingual investigations in the long term.

To reduce the heterogeneity in measures of L2 proficiency, one seemingly easy solution is to rely on the commonly used questionnaires in the field, for example, the language history questionnaire (LHQ, Li et al., 2006, 2014, 2020), the language experience and proficiency questionnaire (LEAP-Q, Marian et al., 2007; Marian & Hayakawa, 2021), and the language and social background questionnaire (LSBQ, Anderson et al., 2018). The published questionnaires present standardised proficiency questions which require participants to rate their L2 proficiency on a Likert scale for each individual skill (e.g., listening, speaking, reading, writing). Admittedly, self-rated proficiency assessment is easy and quick to administer either online or in a paper-and-pencil version, and it can be used in various languages through translation.

Although self-rated proficiency assessment has certain practical advantages, this subjective measure is limited with respect to reliability (i.e., whether it produces consistent results in repeated measures) and validity (i.e., whether it measures what it intends to measure). As shown by Tomoschuk et al. (2019), the correlations between the self-rated English oral proficiency and a standardised picture-naming task (MINT, Multilingual Naming Test, Gollan et al., 2012) interacted with bilingual groups. A closer look at the significant interaction revealed that Spanish–English bilinguals had higher MINT scores than Chinese–English bilinguals despite having the same self-rated speaking proficiency. A subsequent analysis replicated this pattern with speakers of the same language pair such that recently immigrated Chinese–English bilinguals scored lower in the English MINT than bilinguals who grew up in the United States even when their self-ratings were matched. Tomoschuk et al.’s (2019) findings imply that any two bilinguals with identical self-ratings may still differ in L2 proficiency levels. Another alarming finding comes from Lemhöfer and Broersma (2012), who demonstrated that self-ratings of English proficiency obtained with Korean–English bilinguals did not correlate significantly with bilinguals' performance in a standard English test (TOEIC, Test of English for International Communication). Although self-ratings showed significant correlations with objective proficiency measures in other studies (de Bruin et al., 2017; Marian et al., 2007), correlation coefficients varied considerably (e.g., ranging from 0.286 to 0.741 in Marian et al., 2007). Furthermore, self-rated L2 proficiency can be modulated by non-linguistic factors (for further discussion, see Amenta et al., 2020; Brysbaert, 2013; Ferré & Brysbaert, 2017; Izura et al., 2014), such as anxiety levels when using L2 (MacIntyre et al., 1997).

### LexTALE and lextale-type vocabulary tests

Being aware of the potential issues with self-rated proficiency, researchers have striven to develop new tools to objectively assess L2 proficiency. One well-known tool is LexTALE (Lexical Test for Advanced Learners of English) developed by Lemhöfer and Broersma (2012). LexTALE consists of 60 items (i.e., 40 real English words and 20 nonwords) presented in a fixed order to all participants. LexTALE implements an untimed lexical decision task in which participants have to decide whether an item is an English word, without any time limit. To validate LexTALE, Lemhöfer and Broersma correlated the LexTALE scores against bilinguals' performance of the Quick Placement Test (2001) and a translation task. Significant positive correlations were found between the LexTALE scores and the two well-recognised measures of English proficiency, and these findings were consistent for Dutch–English bilinguals and Korean–English bilinguals. Therefore, although LexTALE seems to only tap into participants' word knowledge, its result is a valid proxy for English proficiency. As a valid test, LexTALE takes about 3.5 minutes to complete either online or as a paper-and-pencil test.

In addition to being short and easy to use, LexTALE has several advantages compared to other commonly used proficiency tests. First, in contrast to commercial tests such as the Quick Placement Test, LexTALE is freely available to the research community (included in the published paper and also available at http://www.lextale.com). In comparison to a non-commercial translation task, LexTALE can be used with all English learners, whereas a translation task must be adapted based on participants’ languages (e.g., translating between English and Dutch for Dutch–English bilinguals versus translating between English and Korean for Korean–English bilinguals). There is a non-commercial picture-naming task (i.e., MINT, Gollan et al., 2012), but no paper-and-pencil version exists at the moment that can be easily distributed like LexTALE. Because of its free availability and easy administration, LexTALE has been widely used in bilingual research. In addition to describing bilinguals' English proficiency in an experiment (e.g., Van de Putte et al., 2018), LexTALE has been used as a screening test to select eligible participants (e.g., Declerck et al., 2020), and as an independent variable to investigate participants' performance in a linguistic task (e.g., Diependaele et al., 2013) or a non-linguistic task (e.g., Khare et al., 2013). Consequently, it is advisable to include LexTALE in any research protocol that involves non-native speakers of English, so that readers can compare the proficiency level of participant groups across articles (Brysbaert et al., 2017; Diependaele et al., 2013).

The idea of using LexTALE as the standard in the field clearly converges with the need for improving consistency in L2 proficiency measurements. But this unified approach requires that parallel lexical tests exist for various languages, as L2 differs among bilinguals. Along with the German and Dutch versions of LexTALE provided in Lemhöfer and Broersma (2012), researchers have extended the lextale format to seven other languages, including French (LEXTALE_FR, Brysbaert, 2013), Spanish (Lextale-Esp, Izura et al., 2014), Basque (Basque LexTALE, de Bruin et al., 2017), Italian (LexITA, Amenta et al., 2020), Portuguese (LextPT, Zhou & Li, 2022), Finnish (Lexize, Salmela et al., 2021), and logographical Chinese (LEXTALE_CH, Chan & Chang, 2018). When creating these extensions, researchers had to carefully sample word items based on word frequency to ensure that the extensions match the material used in LexTALE. It is important to note that lextale extensions cannot be developed by simply translating the English word items of LexTALE into another language because word frequencies of translation equivalents do not perfectly correlate (Wen & van Heuven, 2017a). Instead, researchers typically create a lexical test for another language by testing native and non-native speakers with a larger set of items (e.g., 60 words and 60 nonwords in Brysbaert, 2013). When the final set of items are selected (e.g., 56 words and 28 nonwords in Brysbaert, 2013), the test is often administered to a new group of native and non-native speakers in a validation study. Disregarding the different numbers of items included (e.g., 84 items in the French extension vs 90 items in the Spanish extension), the ratio of words versus nonwords remains constant (i.e., 2:1) for all the equivalent lexical tests as well as the original LexTALE.

A difference between the original LexTALE tests developed by Lemhöfer and Broersma (2012) and the subsequent extensions is that authors did not try to equate the difficulty level of the tests across languages (as Lemhöfer and Broersma did for English, German, and Dutch), because there are no agreed standards in developing difficulty-matched tests for various languages (for relevant discussion, see Gollan et al., 2012). In addition, authors often wanted to develop a test that could be used for first language (L1) speakers as well as L2 speakers. Therefore, items were selected so that the best discrimination was possible between the L1 and L2 samples tested, thereby optimising assessment within a language rather than optimising assessment for comparison across languages. Furthermore, the lextale extensions were normally not compared to other well-established measures of proficiency via correlations because validity was considered a given. Therefore, it is important to make a distinction between the original LexTALE tests developed by Lemhöfer and Broersma (2012) for English, German, and Dutch advanced L2 speakers, and the subsequent lextale-type tests developed by others for other languages.

Altogether, lextale-type vocabulary tests in different languages provide an objective estimate of word knowledge. They take less than 5 minutes to complete, and can be used freely. This makes them ideal to combine with other tasks in experimental studies. They allow for direct comparisons of participants within a language, but comparisons between languages should be carefully applied (except for English, German, and Dutch).

### Developing LexCHI

Building on LexTALE and its extensions, the current study presents the development of LexCHI as a lexical test for Chinese using simplified Chinese characters. Simplified Chinese characters are the standardised written form of Chinese in mainland China even though there are various dialects spoken by Chinese speakers (see Gu, 2006, for more information about Chinese dialects). As mentioned above, a lextale-type test has already been created for Chinese using simplified characters (LEXTALE_CH, Chan & Chang, 2018), but this test only includes single characters as items. Testing only single characters to measure Chinese lexical knowledge is a limitation, because most Chinese words contain more than one character. To illustrate this, we analysed 99,121 unique Chinese words from the Chinese subtitle corpus (SUBTLEX-CH, Cai & Brysbaert, 2010; corpus size: 33.5 million words). This revealed that only 5.4% of the words consist of one character, whereas 46.2% of the words have two characters and 24.7% have three characters (12.0% are four-character words and 11.4% are multiple-character words). It is also noteworthy that not all Chinese characters are free morphemes (DeFrancis, 1984; Myers, 2006), so a Chinese character does not always correspond to a word at the lexical level. To create a more appropriate lextale-type test for Chinese, we focused on two-character items for LexCHI, as our analyses showed the majority of Chinese words contain two characters (cf. Li et al., 2015).

In line with lextale-type tests developed for other languages (e.g., Amenta et al., 2020; Izura et al., 2014; Zhou & Li, 2022), the present study develops LexCHI in two studies by testing L1 Chinese speakers as well as L2 Chinese speakers. A preparatory study (Experiment 1) was first conducted to select good items from a larger set of candidates, followed by a validation study (Experiment 2) to test the selected items with a new group of participants. In addition, both studies validated LexCHI akin to LexTALE, such that LexCHI scores would be correlated against a brief version of a standard Chinese proficiency test (i.e., a 20-item cloze test) and a translation task. If LexCHI is a valid test, we would expect significant positive correlations between LexCHI scores and the other two measures of Chinese proficiency. We also tested whether LexCHI is a better lexical test than LEXTALE_CH (Chan & Chang, 2018), as we expected, becauset single-character words are not representative for the full set of Chinese words. To this end, the LEXTALE_CH character test was included in order to compare its correlations with the cloze test and the translation task relative to LexCHI. If LexCHI is indeed a better measure, we would expect higher correlations of LexCHI scores with the other two measurements in comparison with LEXTALE_CH. Including the LEXTALE_CH character test also enables us to explore the relationship between character knowledge and word knowledge. We predicted a significant positive correlation between LEXTALE_CH and LexCHI, because character knowledge is part of word knowledge in Chinese.

### Introducing the normalised Ghent score

In addition to introducing LexCHI, we further propose a new method for scoring the test. It is important to note that LexTALE and its extensions use different equations to correct for the unequal number of word and nonword items presented (e.g., 40 words and 20 nonwords). When developing LexTALE, Lemhöfer and Broersma (2012) recommended calculating the test score as follows:

$$\mathrm{original}\;\mathrm{LexTALE}\;\mathrm{score}=\frac{\mathrm{number}\;\mathrm{of}\;\mathrm{correct}\;\mathrm{words}+2\ast\mathrm{number}\;\mathrm{of}\;\mathrm{correct}\;\mathrm{nonwords}}{\mathrm{number}\;\mathrm{of}\;\mathrm{words}+2\ast\mathrm{number}\;\mathrm{of}\;\mathrm{nonwords}}.$$

Although this calculation showed the strongest correlations with the Quick Placement Test and the translation task, Brysbaert (2013) pointed out that possible scores range between 50% and 100% rather than between 0 and 100% (e.g., if participants respond Yes to all items, this will result in a score of 50%). Brysbaert further suggested computing the Ghent score for the French extension of LexTALE (see Eq. 1).

1 $$\mathrm{Ghent}\;\mathrm{score}={\mathrm N}_{\mathrm{yes}\;\mathrm{to}\;\mathrm{words}}-\frac{{\mathrm N}_{\mathrm{words}}}{{\mathrm N}_{\mathrm{nonwords}}}\times{\mathrm N}_{\mathrm{yes}\;\mathrm{to}\;\mathrm{nonwords}}$$

As indicated in Eq. 1, the Ghent score adjusts Lemhöfer and Broersma's calculation by taking into account the incorrect trials in nonwords instead of the correct ones. Brysbaert's Ghent score has been adopted by follow-up studies, except for the Basque LexTALE. Given that the ratio of words versus nonwords is fixed in all lextale extensions (2:1), the Ghent test score calculation can be simplified to Eq. 2.

2 $$\mathrm{Ghent}\;\mathrm{score}={\mathrm N}_{\mathrm{yes}\;\mathrm{to}\;\mathrm{words}}-2\times{\mathrm N}_{\mathrm{yes}\;\mathrm{to}\;\mathrm{nonwords}}$$

Unfortunately, this approach leads to differences in the score range (e.g., ranging from −56 to 56 in the French extension vs −60 to 60 in the Spanish extension), because the Ghent score range, unlike the original LexTALE score, depends on the number of word and nonword items, which differs across tests (e.g., the number of items is 75, 84 and 90 in the Basque, French and the Spanish tests, respectively). Given the difference in score range, the scoring equation in Lemhöfer and Broersma (2012) is more appealing for studies which use more than one lexical test to measure proficiency in multiple languages at the same time (e.g., de Bruin et al., 2017). Moreover, Lemhöfer and Broersma (2012) recommend a cut-off score that can separate the participants into advanced and intermediate proficiency levels (i.e., C1/C2 vs B2 in the Common European Framework of Reference for Languages), and such cut-off point is not available when using the Ghent score. Therefore, Lemhöfer and Broersma's calculation is also applied when a LexTALE extension is used as a diagnostic tool to filter out participants (e.g., Wen et al., 2021). In view of the pros and cons of the existing equations, we propose the normalised Ghent score, which divides the Ghent score by the number of word items (see Eq. 3).^1^

3 $$\mathrm{normalised}\;\mathrm{Ghent}\;\mathrm{score}=\frac{{\mathrm N}_{\mathrm{yes}\;\mathrm{to}\;\mathrm{words}}-2\times{\mathrm N}_{\mathrm{yes}\;\mathrm{to}\;\mathrm{nonwords}}}{{\mathrm N}_{\mathrm{words}}}$$

The normalised Ghent score has a fixed range of −100% to 100% independent of item numbers, while retaining the advantages of Brysbaert's approach. Furthermore, the normalised Ghent score expressed as percentages is easy to interpret, just like the original LexTALE scoring. Therefore, the normalised Ghent score will be used in the present study.

## Experiment 1: Preparatory study

The primary goal of Experiment 1 is to test a set of 120 items and select a subset of 60 items for LexCHI so that it contains the same number of word and nonword items as the original LexTALE (Lemhöfer & Broersma, 2012). To assess the initial validity, LexCHI scores are correlated with the results of a cloze test and a translation task. We expected significant positive correlations between LexCHI and the cloze test as well as the translation task. Following Lemhöfer and Broersma (2012), we mainly focused on bilinguals who are non-native speakers of Chinese in the correlation analyses, but we also recruited native Chinese speakers for the purpose of item selection (see “Data analysis” for details). Therefore, only non-native Chinese speakers completed the cloze test and the translation task. Additionally, the LEXTALE_CH character test (Chan & Chang, 2018) was included for two reasons. First, it enables a comparison between LexCHI and LEXTALE_CH. For the non-native Chinese speakers, we expected higher correlations for LexCHI scores against the cloze test and the translation task relative to LEXTALE_CH. Second, it enables exploring the relationship between character knowledge and word knowledge. We expected a significant positive correlation between LEXTALE_CH and LexCHI scores. In line with the original LexTALE study (Lemhöfer & Broersma, 2012), a language background questionnaire was included in the experiment to gather detailed information of participants' linguistic profiles.

### Methods

#### Participants

Two groups of participants were recruited for Experiment 1. The first participant group was an L2 group which consisted of 75 non-native speakers of Chinese (female: 54, male: 19, prefer not to say: 2). The L2 participants were recruited at the University of Nottingham Malaysia campus because Malaysia has a considerable number of Chinese learners. They were 24.24 years old on average (range = 18–53, SD = 6.15), and indicated their first language as English (*N* = 60), Malay (*N* = 9), Thai (*N* = 3), Indonesian (*N* = 1), Tamil (*N* = 1), or Vietnamese (*N* = 1). The L2 group received an inconvenience allowance of 10 Malaysian ringgit. Data from seven additional L2 participants were excluded from the analyses due to zero accuracy in the translation task (*N* = 3) or self-reported language impairment (*N* = 4). The second participant group was the L1 group, which consisted of 54 native Chinese speakers (female: 31, male: 23). The L1 participants were recruited online via Prolific (www.prolific.co). They received an inconvenience allowance of £2. L1 participants were 32.2 years old (range = 20–57, SD = 7.62) on average, and they all indicated their first language as Mandarin (*N* = 49) or a Chinese dialect (*N* = 5), e.g., Cantonese. All L1 participants were speakers of Mandarin Chinese (henceforth Chinese). Data from six additional participants were excluded from the analyses due to low self-rated Chinese reading proficiency (< 5 on a 7-point scale, *N* = 3) or because they took an excessively long time to finish the task (> 25 minutes, *N* = 3, possibly due to consulting external tools such as a dictionary). Other language background information of the two participant groups will be presented in the Results section.

#### Procedure and materials

The experiment consisted of a series of tasks presented in a fixed order to participants in Qualtrics. Participants in the L2 group received written instructions in English and completed five tasks, whereas participants in the L1 group received written instructions in Chinese and completed three tasks (Task 1, 2, 5). All participants provided informed consent at the beginning of the study. The study was approved by the Science and Engineering Research Ethics Committee at the University of Nottingham Malaysia Campus (L2 group) in Malaysia and the Ethics Committee at the School of Psychology, University of Nottingham, UK (L1 group).

Task 1: LEXTALE_CH

##### Materials

Chan and Chang (2018) developed a character-based Chinese proficiency test and made it freely available at https://osf.io/qdy4n/. This LEXTALE_CH test consists of 90 items including 30 non-characters and 60 real characters written in simplified Chinese. Among the real characters, 18 have zero occurrences in SUBTLEX-CH (Cai & Brysbaert, 2010), and eight have a frequency less than 1 per million characters. Of the remaining characters, 22 have a frequency ranging from 1 to 10 per million, eight have a frequency between 10 and 100 per million, and four have a frequency higher than 100 per million. The average number of strokes in the 60 characters is 11.5 (range = 4–25).

##### Procedure

The 90 items were presented one at a time in a fixed order to all participants. Participants were instructed to decide whether a presented item was a real Chinese character or not by pressing the Yes or No button on the screen. They were informed that they did not need to respond rapidly, and they should not consult a dictionary. The mean duration of this task was 3.34 minutes for the L2 group (SD = 2.16 minutes) and 2.31 minutes for the L1 group (SD = 1.42 minutes).

##### Scoring

The normalised Ghent scores were calculated for Task 1 using Eq. 3 (60 real characters and 30 noncharacters).

Task 2: LexCHI (120 items)

##### Materials

Similar to LEXTALE_FR (Brysbaert, 2013), 120 items (60 words and 60 nonwords) were selected from a megastudy of simplified Chinese using a lexical decision task (Tsang et al., 2018). All items consisted of two characters with error rates lower than 10% in the megastudy. Following Brysbaert (2013), we selected the same number of word items in each word frequency range. Because a megastudy of simplified Chinese demonstrated that character frequency impacts the recognition of two-character Chinese words (Sun et al., 2018), it is crucial to match character frequency within a word with its word frequency as closely as possible. Therefore, we selected 17 words with a word frequency less than 1 per million (character frequency of each character: < 1 per million), 11 words with a word frequency between 1 and 5 per million (character frequency of each character: 1–5 per million), 16 words with a word frequency between 5 and 10 per million (character frequency of each character: 3–25 per million), nine words with a word frequency between 10 and 20 per million (character frequency of each character: 7–22 per million), 30 words with word frequency between 30 and 100 per million (character frequency of each character: 30–100 per million), and one word with word frequency over 100 per million (character frequency of each character > 100 per million). In order to make nonwords equally difficult, the 60 nonword items (i.e., non-existing sequences of two existing characters) were also selected from the same megastudy by matching character frequency and stroke number with the selected word items. The selected nonwords include two items with a character frequency (for both characters within an item) less than 2 per million, 19 items with a character frequency less than 10 per million, 23 items with a character frequency less than 20 million per million, nine items with a character frequency between 4 and 19 per million, six items with a character frequency between 30 and 100 per million, and one item with a character frequency above 100 per million. There were no repeated characters in words and nonwords. The average stroke number of words and nonwords is 23.18 (SD = 4.87) and 23.47 (SD = 4.47) respectively. It is important to note that the average word frequency of the selected Chinese words is also comparable to that of the English words used in LexTALE (Lemhöfer & Broersma, 2012). Using Zipf values as the standardised word frequency measure (van Heuven et al., 2014), the average word frequency is 3.55 (SD = 0.81) for the selected Chinese words and 3.10 (SD = 0.60) for English words in LexTALE based on the subtitles (Cai & Brysbaert, 2010; van Heuven et al., 2014). The full list of stimuli is provided in Appendix 1.

##### Procedure

The 120 items were presented one at a time in an identical pseudorandom order with words or nonwords occurring no more than five times in a row (Lemhöfer & Broersma, 2012). Participants were instructed to decide whether a presented item was a real Chinese word or not by pressing the Yes or No button on the screen. They were informed that they did not need to respond rapidly, and that they should not consult a dictionary. Similar to Lemhöfer and Broersma (2012), participants were also instructed to press the No button if they were not sure whether an item was a word. The mean duration of this task was 5.25 minutes for the L2 group (5.73 minutes) and 2.34 minutes for the L1 group (SD = 0.74 minutes).

##### Scoring

The normalised Ghent scores were calculated for Task 2 (using the equation in Footnote 1 with 60 words and 60 nonwords).

Task 3: Cloze test

##### Materials

The cloze test consisted of 20 items taken from Hanyu Shuiping Kaoshi (Chinese Proficiency Test). Hanyu Shuiping Kaoshi (HSK) is an official Chinese language proficiency test for non-native Chinese speakers administered by the Confucius Institute Headquarters. HSK provides tests at six levels (i.e., Level 1 to Level 6) for beginning, intermediate and advanced learners. Since a full HSK test takes too long to complete within empirical studies, abridged versions of HSK are often used to measure Chinese proficiency (Li et al., 2019; Zhang et al., 2020). In the reading test of HSK Level 6, there are ten multiple-choice items which require participants to choose one set of correct words from four possible options to complete sentences provided. Such fill-in-the-blank items have been used as cloze tests to measure second language proficiency (Cromheecke & Brysbaert, 2022; Oller, 1973; Tremblay, 2011). The cloze test in our study was not generated by using the sample exam papers provided on the HSK official website because these free resources are easily accessible to participants. Therefore, 20 items were selected from two books which published previous exam papers for HSK Level 6 (Confucius Institute Headquarters, 2016, 2018). The 20 items are composed of six items with three blanks, 12 items with four blanks and two items with five blanks, which mirrors the composition commonly observed in a 10-item set within one HSK test (i.e., including 3 three-blank items, 6 four-blank items and 1 five-blank item). All words presented in the choices consist of two characters. For the words within the correct answers, the average stroke number is 16.18 (SD = 4.79) and the average word frequency is 4.03 (SD = 0.71) in Zipf values (Cai & Brysbaert, 2010; van Heuven et al., 2014).

##### Procedure

Twenty multiple-choice items were presented one by one, and the presentation order was fixed. Participants were instructed to select the correct words that fit in the sentences by clicking on one of the four choices. They could take as long as necessary to make their choice. On average, this task took the L2 group (*N* = 75) 16.00 minutes to complete (SD = 11.44 minutes).

Task 4: Translation task

##### Materials

The translation task consisted of 30 English words and 30 Chinese words selected from an English-Chinese translation database (Wen & van Heuven, 2017a). We first selected 60 English words using the following criteria similar to Lemhöfer and Broersma (2012): (1) the dominant part-of-speech of English words should be a noun; (2) the Chinese–English pairs are non-cognate translations; (3) the English words had no more than three correct Chinese translations (mean = 1.82 , SD = 0.81) and their translation error rates were higher than 50% (mean = 64.94%, SD = 7.51%). Because the translation database of Wen and van Heuven only includes Chinese translations of English words, the most frequent Chinese translations for half of the selected English words were used as the Chinese items to be translated into English. These 30 Chinese items consist of two characters. Their mean number of strokes is 15.57 (SD = 5.24), and their mean word frequency is 3.82 (SD = 0.64) in Zipf values (Cai & Brysbaert, 2010). None of the 30 Chinese items appeared in Task 2. The remaining 30 English words were used as the English items to be translated into Chinese. These English items had a mean word length of 6.33 letters (SD = 1.83) and a mean word frequency of 3.74 (SD = 0.41) in Zipf values (van Heuven et al., 2014). The Chinese and English words used in the translation task are provided in Appendix 2.

##### Procedure

Participants first completed the English-to-Chinese translation task and then the Chinese-to-English translation task. English or Chinese items were presented one at a time, and all items were presented in a fixed order. Participants were required to provide the first Chinese/English translation that came to their mind (Tokowicz & Kroll, 2007; Wen & van Heuven, 2017a; Wu & Thierry, 2010). They could take as long as necessary to type in their answers, and they were asked to skip an item by pressing the Next button on the screen if they could not provide a translation. On average, this task took the L2 group (*N* = 75) 13.14 minutes to complete (SD = 7.92 minutes).

##### Scoring

The translations provided by the participants were first automatically compared to the correct translation included in the English-Chinese translation database (Wen & van Heuven, 2017a). All other translations were manually checked using the Oxford Advanced Learner's English-Chinese Dictionary (Hornby, 2018). Responses with typos or spelling mistakes were scored as incorrect.

Task 5: Language background questionnaire

##### Materials

The aim of the language background questionnaire is to understand participants' experience with Chinese language (e.g., age of first contact, years of experiences, the language used by parents or carers during childhood, the main instruction language used by teachers from kindergarten to university) and their self-perceived Chinese proficiency. The questions were adapted from the questionnaire used in prior work to obtain participants' linguistic profile (e.g., Wen et al., 2018; Wen & van Heuven, 2017a, 2018). For example, participants were asked to rate their ability of Chinese speaking, listening, reading and writing ability separately on a 7-point scale (1 = very poor, 7 = native-like) by receiving the following question, i.e., "Indicate how good you consider yourself in listening, speaking, reading, and writing in Chinese (Mandarin)".

### Data analysis

A series of correlation analyses were conducted separately for the L1 and L2 groups. Spearman's rank correlation coefficients were calculated when involving self-rating data, and Pearson correlation coefficients were calculated when rating data were not involved.

Following recent studies (e.g., Amenta et al., 2020; Brysbaert, 2013; Izura et al., 2014), a two-step analysis was conducted to evaluate the items. In the first step, the point-biserial correlation was calculated. In the second step, the item response theory analysis was conducted using the ltm package (Rizopoulos, 2006) in R version 4.1.0 (R Core Team, 2021). In both steps, data of the L1 and L2 group were combined in the analyses, but the analyses were conducted separately for word and nonword items. Thus, a point-biserial correlation was first calculated between the participants' accuracy of one word/nonword item and their overall accuracy across all word/nonword items. Like the Pearson correlation coefficient, the point-biserial correlation coefficient also ranges between −1 and 1. For a word item, a positive correlation indicates that participants who correctly identify the word are likely to obtain higher scores for the word items. Likewise, a positive correlation for a nonword item suggests that participants who correctly reject the nonword are likely to have higher scores. Thus, for both word and nonword items, positive correlations are the first criterion for good items, and items with negative correlations should be deleted before running the item response theory analysis. In the second step, the item response theory analysis was conducted in which a latent variable modelling was run separately for words and nonwords (see Şahin & Anil, 2017, for discussion about sample sizes in the IRT analysis). Each model produced two values for all items, i.e., difficulty and discriminative power. Following previous studies (Amenta et al., 2020; Chan & Chang, 2018; Izura et al., 2014), the selected items should vary in terms of difficulty and have a good discriminative power, so the selection was based on both difficulty and discriminative power parameters. Therefore, word and nonword items were separately ordered based on the difficulty parameter and were grouped into 20 groups (three items per group). Within each group, two words or one nonword with highest discriminative parameter were then selected given that we aimed to select 40 word items and 20 nonword items for the final set of LexCHI.

### Results

The results of each experimental task are shown in Table 1, and details of participants' language background obtained from the language background questionnaire (Task 5) are summarised in Table 2.

**Table 1:** Results of four experimental tasks in Experiment 1 (%, with SD in brackets)

	Mean (SD)	
	L2 group (*N* = 75)	L1 group (*N* = 54)
Task 1: LEXTALE_CH	47.22 (20.50)	68.80 (9.73)
Task 2: LexCHI	43.62 (29.02)	91.70 (13.16)
Task 3: Cloze	63.13 (20.89)	
Task 4: Translation (Overall)	47.36 (22.11)	
Task 4: Translation (E–C)	42.62 (21.29)	
Task 4: Translation (C–E)	52.09 (24.43)	

The maximal score of all tasks is 100%. Translation (E–C) is translating from English to Chinese, and translation (C–E) is translating from Chinese to English

**Table 2:** Summary of participants' language background data from both groups in Experiment 1

	Mean (SD)	
	L2 group (*N* = 75)	L1 group (*N* = 54)
Age exposed to Chinese (years)	4.68 (3.70)	
Experience with Chinese (years)	17.16 (7.44)	
Average self-rated Chinese ability	4.16 (0.97)	6.55 (0.74)
Self-rated Chinese ability (Listening)	4.93 (0.95)	6.77 (0.57)
Self-rated Chinese ability (Speaking)	4.65 (1.16)	6.56 (0.88)
Self-rated Chinese ability (Reading)	3.77 (1.23)	6.69 (0.64)
Self-rated Chinese ability (Writing)	3.27 (1.33)	6.17 (1.41)

Subjective Chinese (Mandarin) ability were rated on a 7-point scale (1 = very poor, 7 = native-like). Average self-rated Chinese ability is the mean of self-rated Chinese ability of listening, speaking, reading and writing

#### Correlations

Table 3 shows the correlations of the normalised Ghent scores of LEXTALE_CH (Task 1) and LexCHI (Task 2, 120 items) against the accuracy rates of the cloze test (Task 3), the translation task (Task 4), and self-ratings of Chinese ability for the L2 group. As Table 3 shows, LexCHI and LEXTALE_CH significantly correlated with all the other measures, and both tests had the highest correlation with the cloze test (LexCHI: *r* = 0.81, *p* < .001, the LEXTALE_CH: *r* = 0.68, *p* < .001). When the correlations of LexCHI with the cloze test and self-ratings were compared to those of LEXTALE_CH, LexCHI consistently outperformed LEXTALE_CH. In addition, LEXTALE_CH and LexCHI positively correlated (*r* = 0.72, *p* < .001).

**Table 3:** Correlations of LEXTALE_CH (Task 1) and LexCHI (Task 2) against the cloze test (Task 3), the translation task (Task 4) and the self-ratings of Chinese ability (L2 group in Experiment 1, *N* = 75)

	LEXTALE_CH	LexCHI
Task 3: Cloze test	0.68***	0.81***
Task 4: Translation	0.62***	0.61***
Average self-rated Chinese ability	0.43***	0.57***
Self-rated Chinese ability (Listening)	0.26*	0.40***
Self-rated Chinese ability (Speaking)	0.26*	0.36**
Self-rated Chinese ability (Reading)	0.45***	0.60***
Self-rated Chinese ability (Writing)	0.47***	0.55***

* *p* < .05, ** *p* < .01, *** p < .001

Table 4 shows the correlations of the normalised Ghent scores of the LEXTALE_CH (Task 1) and LexCHI (Task 2, 120 items) against all the self-ratings of Chinese ability for the L1 group. As can been seen in Table 4, LexCHI significantly correlated with all the self-ratings of Chinese ability whereas LEXTALE_CH only significantly correlated with the average self-rating across four skills and the self-rated writing ability. Consistent with the patterns observed in the L2 group, higher positive correlations were found between LexCHI and the self-ratings than between the character task and the self-ratings. For the L1 group, LEXTALE_CH and LexCHI also significantly correlated (*r* = 0.62, *p* < .001).

**Table 4:** Correlations of LEXTALE_CH (Task 1) and LexCHI (Task 2) against the self-ratings of Chinese ability (L1 group, Experiment 1, *N* = 54)

	LEXTALE_CH	LexCHI
Average self-rated Chinese ability	0.31*	0.50***
Self-rated Chinese ability (Listening)	0.01	0.33*
Self-rated Chinese ability (Speaking)	0.25^+^	0.36**
Self-rated Chinese ability (Reading)	0.26^+^	0.46***
Self-rated Chinese ability (Writing)	0.36**	0.53***

^+^*.*10 *> p* > .05, * *p* < .05, ** *p* < .01, *** p < .001

#### Item selection for LexCHI

**Fig. 1:**
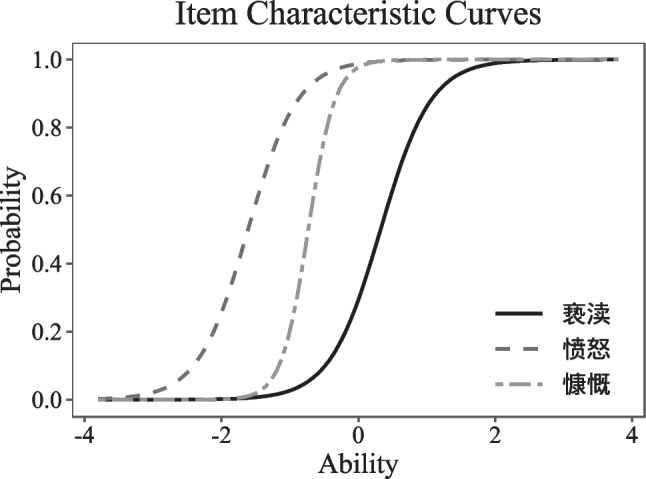
Item response curves for three Chinese word: 亵渎 (profanity), 愤怒 (anger) and 慷慨 (generous)

For the point-biserial correlation analyses, all the word and nonword items showed positive correlations (range: 0.29–0.75 for words, 0.30–0.71 for nonwords). Therefore, all 60 words and 60 nonwords were included in the item response theory analysis. Figure 1 illustrates the results of three-word items in the item response theory analysis. In Fig. 1, the difficulty (ability) parameter is represented on the *x*-axis, and each item's difficulty value is the *x* value when its curve reaches 0.5 on the *y*-axis (the dotted line). Thus, the word 亵渎 (*profanity*, difficulty = 0.32) is more difficult than the word 慷慨 (*generous*, difficulty = −0.73) and 愤怒 (*anger*, difficulty = −1.61). On the other hand, the discriminative parameter is represented by the steepness of the curves. Thus, the word 慷慨 (*generous*, discriminative = 5.14) has better discriminative power than the word 亵渎 (*profanity*, discriminative = 2.71) and 愤怒 (*anger*, discriminative = 2.76). Based on the results of the item response theory analysis (see “Data analysis” for the detailed selection procedure), 40 words and 20 nonwords were selected for the final version of LexCHI (see Appendix 3 for the full list of items). The lexical characteristics of selected items are summarised in Table 5.

**Table 5:** Lexical characteristics of selected items (with ranges in brackets)

	Word frequency per million	Zipf value	Stroke number	Character frequency per million (first character)	Character frequency per million (second character)
Words (*N* = 40)	1242.72 (2–33364)	3.63 (1.95–6.00)	22.73 (14–37)	48.48 (0.09–1374.33)	32.84 (0.06–801.09)
Nonwords (*N* = 20)	NA	NA	22.60 (16–29)	10.29 (0.38–86.80)	8.584 (0.130–47.86)

### Discussion

In Experiment 1, we set out to test 120 two-character Chinese items and select 60 items with varying difficult levels and good discriminative power for LexCHI. In addition to the 120-item LexCHI, we also administrated a cloze test and a translation task to a group of non-native Chinese speakers (L2 group). The L2 group's performance in the cloze test and the translation task was correlated against LexCHI as a means of accessing its initial validity. As expected, significant positive correlations of LexCHI against the cloze test and the translation task were found, providing evidence for the LexCHI score as a valid index of Chinese proficiency. Also, the correlation of the cloze test was higher for LexCHI than LEXTALE_CH, indicating that LexCHI is a better lexical test for estimating Chinese proficiency. For both L1 and L2 groups, LexCHI significantly correlated with all the self-ratings of Chinese proficiency, with higher correlations in the L2 group. This pattern is in line with previous studies which reported that correlations between objective and subjective measures of proficiency were higher in the weaker language than in the stronger language of bilinguals (Gollan et al., 2012; Marian et al., 2007; Sheng et al., 2014). As predicted, LexCHI and LEXTALE_CH positively correlated in both groups. Importantly, the LexCHI data of the L1 and L2 groups were combined in a two-step analysis to select 60 good items out of the 120-item set. Based on the point-biserial correlation and the item response theory analysis, 40 word items and 20 nonword items were selected.

Like other lextale extensions (e.g., Amenta et al., 2020; Izura et al., 2014; Zhou & Li, 2022), the final set of items in LexCHI would be evaluated with a new group of native and non-native speakers of Chinese in Experiment 2. Experiment 2 also includes the LEXTALE_CH character test, a cloze test and a translation task. Note that five participants removed from the L1 group in Experiment 1 indicated English or Spanish as their first language even though demographic filters in Prolific were applied to target L1 Chinese speakers only. These five participants could not be included as L2 participants because they did not take the cloze test and the translation task. Therefore, the same set of tasks are administered to both L1 and L2 groups in Experiment 2 so that participants could be assigned to a different group if needed.

## Experiment 2: Validation study

Experiment 2 was used to further evaluate validity and reliability of the final set of 60 items in LexCHI. This evaluation was conducted with new groups of native and non-native Chinese speakers. Similar to Experiment 1, Experiment 2 included LEXTALE_CH (Chan & Chang, 2018), a cloze test, a translation task and a language background questionnaire. Because most non-native Chinese speakers in Experiment 1 indicated English as their first language, we decided to recruit non-native Chinese speakers with English as their first language for the L2 group in Experiment 2. LexTALE (Lemhöfer & Broersma, 2012) was also included to obtain an objective English proficiency measure. L2 participants should have high scores in the English vocabulary test (LexTALE) because their L1 is English even though they might have low scores in the tasks involving Chinese (e.g., LexCHI). In line with Experiment 1, we predicted significant correlations of LexCHI scores with the cloze test and the translation task in the L2 group. Furthermore, a higher positive correlation for LexCHI scores than the LEXTALE_CH scores is expected when using the cloze test and the translation task as the reference. Finally, we also expected a significant positive correlation between the LEXTALE_CH and LexCHI.

### Methods

#### Participants

A group of non-native Chinese speakers (L2 group) and a group of native Chinese speakers (L1 group) were recruited online via Prolific (seven participants swapped between groups).^2^ All participants received an inconvenience allowance of £6. The L2 group consisted of 59 non-native Chinese speakers (female: 32, male: 27; age: mean = 26.58 years, range = 18–44, SD = 6.07). The L2 participants indicated either English (*N* = 44) or Chinese (*N* =15) as the first language. Although 15 participants of the L2 group indicated Chinese (Mandarin or a Chinese dialect, e.g., Shanghainese, Cantonese, Hakka) as the first language which was used by their parents/carers during childhood, most of these participants received all their education in English (*N* = 12; for one participant, Mandarin was used as the main language for instructions only during secondary education; for two participants, Mandarin or Cantonese was used the main language for instructions only in kindergarten). These participants did not speak other languages in addition to English and Chinese (Mandarin or a Chinese dialect). Their self-ratings of English proficiency for four skills (listening, speaking, reading, writing) were all 7 (i.e., native-like), whereas their average self-rated Chinese proficiency (the mean of four skills) was 3.20 (range = 1–5.75) on a 7-point scale.^3^ Therefore, these 15 participants should be considered as non-native Chinese speakers. Data from 18 additional participants were excluded from the analyses due to bad performance in the English vocabulary test (LexTALE scores < 80, see Footnote 2, *N* = 6), zero accuracy in the translation task (*N* = 3), using traditional Chinese characters in the translation task (*N* = 7) or self-reported language impairment (*N* = 2). The L1 group consisted of 46 native Chinese speakers (female: 23, male: 23; age: mean = 30.80 years, range = 19–60, SD = 9.15). All L1 participants indicated Mandarin (*N* = 43) or a Chinese dialect (*N* = 3) as their first language and were speakers of Mandarin Chinese. Data from four additional participants were excluded from the analyses due to low self-rated Chinese reading proficiency (< 5 on a 7-point scale, *N* = 1),^4^ zero accuracy in the translation task (*N* = 1) or using traditional Chinese characters in the translation task (*N* = 1). Additional language background information of the two participant groups will be presented in the Results section.

#### Procedure, materials and scoring

The experiment consisted of six tasks presented in a fixed order to participants in Qualtrics. Written instructions were provided in English for both groups. All participants gave informed consent at the beginning of the study. The study was approved by the Ethics Committee of the School of Psychology at the University of Nottingham. The overall procedure was identical to Experiment 1 (the L2 group) except for two aspects. First, there were 60 items in Task 2 (LexCHI) instead of 120 items. On average, this task now took 2.06 minutes to complete (SD = 2.49 minutes). Second, before the final language background questionnaire, an English vocabulary test (LexTALE, Lemhöfer & Broersma, 2012) was included as Task 5. This task consisted of 40 real English words and 20 nonwords which were presented one at a time in a fixed order to all participants. Participants were instructed to decide whether a letter string was an English word or not by pressing the Yes or No button on the screen. They were informed that they did not need to respond rapidly, and they should not consult a dictionary. On average, this task took 1.97 minutes to complete (SD = 1.69 minutes).

The normalised Ghent scores were calculated for Task 1, Task 2 and Task 5 using Eq. 3.

### Data analysis

A series of correlation analyses were conducted separately for the L1 and L2 groups. Pearson correlation coefficients were calculated except that Spearman's rank correlation coefficients were computed for data involving self-ratings. To evaluate the final set of 60 items for LexCHI, the reliability of 60 items was measured with Cronbach's alpha and the split-half correlation with the help of the ltm package (Rizopoulos, 2006) and the performance package (Lüdecke et al., 2021) in R version 4.1.0 (R Core Team, 2021). Data of the L1 and L2 groups were combined in the reliability analysis.

### Results

The results of each experimental task are shown in Table 6, and details of participants' language background obtained from the language background questionnaire (Task 6) are summarised in Table 7. As can be seen from Table 6, the L1 group outperformed the L2 group in all tasks involving Chinese (Task 1–4). Critically, for the 60-item LexCHI (Task 2), the normalised Ghent scores of the L2 group were significantly lower than those of the L1 group (Wilcoxon rank-sum test, W = 2644, *p* < .001, effect size *d* = −3.23).

**Table 6:** Results of four experimental tasks in Experiment 2 (%)

	Mean (SD)		Effect size [95% CI]
	L2 group (*N* = 59)	L1 group (*N* = 46)	
Task 1: LEXTALE_CH	24.41 (25.34)	69.57 (7.18)	−2.30 [−2.8, −1.81]
Task 2: LexCHI	13.05 (31.95)	93.10 (9.46)	−3.23 [−3.81, −2.65]
Task 3: Cloze test	44.15 (25.09)	95.22 (6.83)	−2.64 [−3.16, −2.11]
Task 4: Translation task (Overall)	42.88 (28.06)	75.94 (19.59)	−1.34 [−1.76, −0.91]
Task 4: Translation task (E–C)	36.27 (31.52)	72.10 (23.72)	−1.26 [−1.68, −0.84]
Task 4: Translation task (C–E)	49.49 (31.24)	79.78 (18.70)	−1.14 [−1.56, −0.73]
Task 5: LexTALE	89.19 (9.60)	50.00 (25.85)	2.11 [1.63, 2.59]

The maximal score of all tasks is 100%. Effect size (*d*) and its 95% confidence intervals were calculated using https://www.campbellcollaboration.org/escalc/html/EffectSizeCalculator-SMD1.php. Translation task (E–C) is translating from English to Chinese, and translation task (C–E) is translating from Chinese to English

**Table 7:** Summary of participants' language background data from both groups in Experiment 2

	Mean (SD)	
	L2 group (*N* = 59)	L1 group (*N* = 46)
Age exposed to Chinese (years)	4.93 (6.85)	
Experience with Chinese (years)	14.25 (10.37)	
Average self-rated Chinese ability	3.73 (1.20)	6.53 (0.97)
Self-rated Chinese ability (Listening)	4.88 (1.42)	6.78 (0.73)
Self-rated Chinese ability (Speaking)	4.22 (1.60)	6.63 (0.90)
Self-rated Chinese ability (Reading)	3.19 (1.53)	6.61 (1.02)
Self-rated Chinese ability (Writing)	2.61 (1.51)	6.11 (1.51)

Subjective Chinese (Mandarin) ability was rated on a 7-point scale (1 = very poor, 7 = native-like). Average self-rated Chinese ability is the mean of self-rated Chinese ability of listening, speaking, reading and writing

#### Correlations

Table 8 shows the correlations of the normalised Ghent scores of LEXTALE_CH (Task 1) and LexCHI (Task 2, 60 items) against the accuracy rates of the cloze test (Task 3), the translation task (Task 4), and self-ratings of Chinese ability.

**Table 8:** Correlations of LEXTALE_CH (Task 1) and LexCHI (Task 2, 60 items) against the cloze test (Task 3), the translation task (Task 4) and the self-ratings of Chinese ability (Experiment 2)

	L2 group (*N* = 59)		L1 group (*N* = 46)	
	LEXTALE_CH	LexCHI	LEXTALE_CH	LexCHI
Cloze test	0.45***	0.47***	0.41**	0.35*
Translation	0.47***	0.61***	0.12	0.16
Average self-rated Chinese ability	0.37**	0.40**	0.34*	0.23
Self-rated Chinese ability (Listening)	0.11	0.16	0.25^+^	0.22
Self-rated Chinese ability (Speaking)	0.17	0.20	0.29^+^	0.26^+^
Self-rated Chinese ability (Reading)	0.51***	0.49***	0.23	0.17
Self-rated Chinese ability (Writing)	0.36**	0.39**	0.36*	0.26^+^

^+^10 *> p* > .05, * *p* < .05, ** *p* < .01, *** p < .001. See Appendix 4 for the correlation results when the L1 and L2 groups are combined

**Fig. 2:**
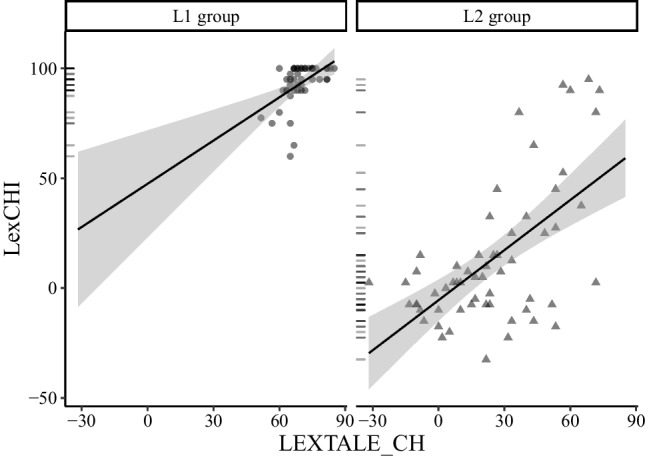
LexCHI by LEXTALE_CH in Experiment 2 with a linear regression line which is shaded by the 95% confidence intervals (normalised Ghent scores; Left: L1 group; Right: L2 group)

For the L2 group, Table 8 shows that both LEXTALE_CH and LexCHI correlated significantly with the cloze test and the translation task. Compared with LEXTALE_CH, LexCHI revealed slightly higher correlations with the cloze test, the translation task and all the self-ratings of Chinese proficiency except for the self-rated reading proficiency. Both LEXTALE_CH and LexCHI had the highest correlation with the translation task. Additionally, LEXTALE_CH and LexCHI were positively correlated (*r* = 0.60, *p* < .001, see Fig. 2).

Results of the L1 group revealed that LEXTALE_CH and LexCHI significantly correlated with the cloze test, but not the translation task. As can be seen in Table 8, there are also significant correlations of LEXTALE_CH with the average self-rating (across four skills) and the self-rated writing ability. Assuming that the translation task should be correlated with participants' L2 (English) proficiency, we also checked the relationship between the translation task and LexTALE, which indeed revealed a significant correlation, *r* = 0.37, *p = *.011. LEXTALE_CH and LexCHI also positively correlated (*r* = 0.50, *p* < .001, see Fig. 2).

#### Reliability (LexCHI)

The Cronbach's alpha for the 60-item LexCHI was 0.96, which indicates a high reliability of the test. The high Cronbach's alpha obtained for LexCHI was very similar to that of other versions of LexTALE, e.g., 0.96 for the Italian version (Amenta et al., 2020), 0.96 for the French version (Brysbaert, 2013), 0.95 for the character-based Chinese version (Chan & Chang, 2018). In line with Cronbach's alpha, the split-half correlation was very high (*r* = .922, the Spearman-Brown corrected correlation: *r* ﻿= .959)﻿.^5^

### Discussion

Experiment 2 evaluated the final version of LexCHI with a new group of participants. The first key finding is that LexCHI with the 60 items has a high reliability (e.g., Cronbach's alpha = 0.96). Another important finding is that LexCHI scores of L1 participants were significantly higher than those of the L2 participants (effect size *d* = −3.23). As predicted, LexCHI scores of the L2 group significantly correlated with the cloze test and the translation task, thus mirroring the findings in Experiment 1. This consistent finding clearly demonstrated the validity of LexCHI. Another finding consistent with Experiment 1 is that LexCHI and the LEXTALE_CH character test positively correlated (see Fig. 2). In line with our prediction for the L2 group, LexCHI correlations with the cloze test and the translation task were higher than those for LEXTALE_CH with the cloze test and translation task. This result also replicated the findings of Experiment 1. Because LexCHI consistently outperformed LEXTALE_CH in the L2 groups, LexCHI is a better lexical test for estimating Chinese proficiency of non-native speakers.

To further explore whether LEXTALE_CH and LexCHI measure different aspects of Chinese knowledge, we conducted a post-hoc regression analysis on the combined data of L1 and L2 groups with LEXTALE_CH and LexCHI as predictors. Accuracy of the cloze test was chosen as the dependent variable in the regression analysis because both LEXTALE_CH and LexCHI had higher correlation with the cloze test than with the translation task when the data of L1 and L2 groups were combined (see Appendix 4). To address the issue of the collinearity between the two predictors, the scores of LEXTALE_CH were first orthogonalised by fitting a linear model in which LEXTALE_CH scores were predicted by the LexCHI scores (see Wen & van Heuven, 2017a, for a similar approach). The residuals of this model were to be used in the regression analysis as the predictor of LEXTALE_CH. In the regression analysis, the LexCHI scores were entered in the first step to predict accuracy of the cloze test, and the LEXTALE_CH scores were entered in the second step (see Appendix 4 for the exploration with the LEXTALE_CH scores entered first). In the first step, LexCHI was a significant predictor (β = 0.56203, SE = 0.03782, *t* = 14.86, *p* < .001; *R*^2 = ^0.682, adjusted *R*^2 = ^0.6789). In the second step, LexCHI and LEXTALE_CH were both significant predictors (LexCHI: β = 0.56203, SE = 0.03661, *t* = 15.350, *p* < .001; LEXTALE_CH: β = 0.30580, SE = 0.10888, *t* = 2.809, *p* < .01; *R*^2 = ^0.7048, adjusted *R*^2 = ^0.699). Although the regression analysis showed that participants' scores in both LexCHI and LEXTALE_CH significantly predicted their performance of the cloze test, it is clear that LEXTALE_CH scores only accounted for a small portion of the variance in the cloze test accuracy as indicated by a less-than-3% increase of *R*^2^ in the second step. Taken together, it is likely that LEXTALE_CH is able to provide certain complementary information of participants' Chinese knowledge on top of LexCHI. However, further research is needed to find out what kind of additional knowledge can be measured by LEXTALE_CH. We recommend using both tests when researchers need to have a fine-grained description of participants' Chinese knowledge and using LexCHI alone when a valid proficiency measure of Chinese is sufficient.

## General discussion

The present study was designed to develop LexCHI, a Chinese extension of LexTALE (Lemhöfer & Broersma, 2012). In two experiments, we evaluated LexCHI and demonstrated that this lexical test is a good indicator of Chinese proficiency. As a valid lexical test, LexCHI will be a useful instrument for researchers interested in Chinese processing by second language Chinese speakers.

When studying Chinese processing in non-native speakers (e.g., Chang et al., 2015; Chen et al., 2018; Li et al., 2019; Pelzl et al., 2021), researchers need to measure Chinese proficiency, because it is common practice to report language proficiency in non-native speakers (see Zhang, 2018, for a review of research on Chinese learning which revealed that most studies did not report learners’ Chinese proficiency). Because participants in a study often have not taken a standard Chinese proficiency test (e.g., HSK), measures of language proficiency need to be gathered during an experiment. Even if it is possible to recruit participants who have taken a standard Chinese proficiency test, their test scores could be several months or years old, and therefore such scores do not represent their current proficiency because language proficiency is likely to change over time. When a freely available Chinese test is absent, researchers opt to either create an abridged version of a commercial proficiency test (e.g., HSK) which unavoidably varies across research groups and cannot be publicly shared as restricted by copyright, or simply rely on self-rated proficiency. Although self-rated proficiency is certainly better than no measures at all, subjective assessment is clearly inferior to objective assessment as discussed in the Introduction. The existing LEXTALE_CH character test (Chan & Chang, 2018) is not a widely used measure of Chinese proficiency, which may be attributed to the concern that it only measures character knowledge. Instead, LexCHI, like LexTALE and its extensions, is a short vocabulary test to measure language proficiency. The use of standardised lexical tests such as LexCHI will minimise discrepancies in proficiency measurements in future studies involving bilingual populations.

A lexical test such as LexCHI that assesses vocabulary knowledge provides, unfortunately, an assessment of only one dimension within the multidimensional construct of language proficiency. However, assuming vocabulary knowledge as a snapshot of proximate proficiency not only converges with researchers' intuition but also is supported by empirical evidence. In particular, a meta-analysis of 126 studies by Zhang and Zhang (2020) revealed that vocabulary tests correlated well with L2 speakers' performance in reading comprehension (*r* = .57, *p* < .01) and listening comprehension (*r* = .56, *p* < .01). Nevertheless, one may still argue that lexical knowledge expressed as performance in a vocabulary test is not a perfect index of proficiency. Unfortunately, there is currently no existing gold standard test for measuring proficiency because no single test can capture all aspects of language proficiency. What is crucial is to provide researchers with a valid and sensitive measurement that is feasible in experimental settings to estimate language proficiency. Therefore, it is highly advisable to include LexTALE and its extensions in bilingual studies to objectively measure proficiency. Meanwhile, language proficiency questions presented in questionnaires should not be totally avoided. While self-rated L2 proficiency is not the optimal tool for measuring proficiency levels, self-ratings assess participants' perceived proficiency and can provide useful supplementary information of linguistic profiles (Gollan et al., 2012). The issue lies in using the subjective measure as the only index of proficiency (see de Bruin, 2019; Luk & Bialystok, 2013; Prior & van Hell, 2021, for the call for objectively measuring proficiency apart from subjective assessment).

In addition to developing LexCHI and providing the test freely for researchers, the present study contributes to the field by proposing the normalised Ghent score. As mentioned earlier, the normalised Ghent score is based on the equations introduced by Lemhöfer and Broersma (2012) and Brysbaert (2013). It is noteworthy that the normalised Ghent score is a linear equivalent of its predecessors. Therefore, correlation analyses involving the normalised Ghent score presented here do not change when using previous scoring equations. Compared with its predecessors, the normalised Ghent score takes into account the need for a fixed range of possible scores (ranging from −100% to 100%) and the participants' tendency to adopt a guessing strategy. For example, Table 9 shows that a participant providing a unique response to all items (all Yes or all No) will have a score of 0.

**Table 9:** Possible scores in extreme situations

Correct trials in words	Correct trials in nonwords	Normalised Ghent score (%)
40	20	100
40	0	0
0	20	0
0	0	−100

**Fig. 3:**
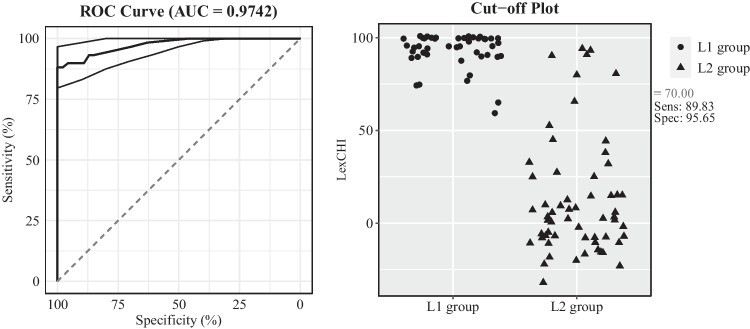
Receiver operator characteristic (ROC) curve with the 95% confidence intervals (left) and optimal criterion cut-off plot (right) for LexCHI (normalised Ghent scores, %)

As mentioned in the Introduction, Lemhöfer and Broersma (2012) recommend a cut-off score that can classify participants into different proficiency levels (intermediate vs advanced). To provide a similar cut-off point for LexCHI using the normalised Ghent scores, a receiver operator characteristic (ROC) curve analysis was conducted (Lalkhen & McCluskey, 2008; Read et al., 2015). The ROC curve analysis has been used widely in clinical areas to evaluate how accurate a diagnostic test is in classifying two populations (e.g., people with or without dyslexia). The ROC curve analysis calculates the area under the curve (AUC) as a measure of discrimination power (e.g., > .80 means good discrimination, maximal value: 1), as well as the sensitivity (e.g., how accurate the test is in identifying people with dyslexia) and the specificity (e.g., how accurate the test is in distinguishing people without dyslexia) of a cut-off value. The results of our ROC curve analysis using the data of the L1 group and the L2 group in Experiment 2 are plotted in Fig. 3. As can be seen in the left panel, the AUC value is near perfect (i.e., 0.974), which converged with the significant differences in LexCHI scores between the L1 and L2 participants in Experiment 2. Furthermore, the cut-off score of 70% has high sensitivity (i.e., 0.957) and specificity (i.e., 0.898) values.^6^ In light of these results, if a participant has a score lower than 70%, it is very likely that this participant is not a native speaker of Chinese. This cut-off score can not only be used as a threshold to identify bilinguals with a native-like proficiency of Chinese (e.g., the C2 level in the Common European Framework of Reference for Languages) but also as a filter to screen native Chinses speakers. It is necessary to have a screening test when recruiting native Chinese speakers for an online study because we found out that applying demographic filters in Prolific not always results in obtaining native speakers of a given language (see the Discussion in Experiment 1 and Footnote 2). A possible explanation is that participants might have different interpretations of the term native/first language. Moreover, participants may indicate Chinese as the first language, but they do not obtain native-like level of proficiency as they have been immersed in a non-Chinese-speaking environment from early childhood (i.e., Chinese heritage speakers). Taken together, LexCHI can used as a screening test to identify participants with native or native-like level of proficiency.

Overall, LexCHI is a valuable tool for the research community because it also opens the door to new avenues for further research. For instance, LexCHI can be used in megastudies of Chinese (e.g., Sze et al., 2015; Tsang et al., 2018; Tse et al., 2016) to explore individual differences in first language processing (Andrews et al., 2018; Andrews & Lo, 2012; Beyersmann et al., 2015; see Kidd et al., 2018 for a review). Another potential use is to employ LexCHI as part of a battery test and generate proficiency norms for native speakers (e.g., Dujardin et al., 2022). Such a proficiency norm could be used as an indicator for participants with reading difficulties (e.g., dyslexia). In terms of bilingual investigations, proficiency of bilinguals' both languages can be measured with LexCHI and other similar lexical tests, and the two test scores can be used as a composite measure to objectively determine the stronger (i.e., dominant) language of a bilingual (e.g., Gollan et al., 2012; Sheng et al., 2014; Tomoschuk et al., 2019).

In anticipation of future research making the most of LexCHI, we would like to emphasise that researchers should be cautious in choosing LexCHI over other valid Chinese proficiency tests, e.g., a Chinese C-test (Malone & Xu, 2019), the Chinese version of the MINT (Multilingual Naming Test, Gollan et al., 2012) and the LEXTALE_CH character test (Chan & Chang, 2018). As Hulstijn (2012) points out, the choice of objective tests should be justified in an experiment by the research purpose. For example, in the case of an experiment with a naming task, a standardised picture-naming test like the MINT (Gollan et al., 2012) may be a better predictor of experimental performance relative to LexCHI. Similarly, for a study on Chinese character recognition, LEXTALE_CH (Chan & Chang, 2018) may outperform LexCHI in accounting for differences in the experimental task. Moreover, a limitation of LexCHI is that the test involves simplified Chinese characters and not traditional Chinese characters. Given this limitation, LexCHI scores of native or non-native Chinese speakers who use traditional Chinese characters (e.g., people living in Hong Kong and Taiwan) should be interpreted with caution. In addition, like other lextale-type vocabulary tests (e.g., Brysbaert, 2013), LexCHI was developed for native/non-native adult speakers, and thus it is unclear whether LexCHI is suitable to measure Chinese proficiency of children. To measure Chinese proficiency among non-adult native speaker of Chinese, a recently published vocabulary test may be more appropriate since this freely available test was developed with middle/high-school students who are native speakers of Chinese (Qi et al., 2022). In brief, the practice of opting for a proficiency measurement that is convenient without justifications should be avoided because there is no one-size-fits-all measure of proficiency.

To summarise, the present study introduced LexCHI as a valid lexical test to measure Chinese proficiency. LexCHI consists of 40 words and 20 nonwords presented in simplified Chinese and implements an untimed lexical decision task. On average, it takes less than 3 minutes to complete LexCHI. This short test can be easily distributed as a paper-and-pencil test (items available in supplementary materials and on the Open Science Framework, and instructions in English or Chinese are also available on the Open Science Framework, https://osf.io/dh3ty/) or included in an online study (all items in png format can also be downloaded from the Open Science Framework). For the scoring of LexCHI, our normalised Ghent score is recommended. Because LexCHI is a Chinese extension of the widely used LexTALE, LexCHI also contributes to the endeavours towards reliably and effectively measuring proficiency in experimental settings, which aims to eliminate between-study variability in proficiency measures. As a useful tool, LexCHI can be further applied in new avenues for further research.

## Figures and Tables

**Fig. 4:**
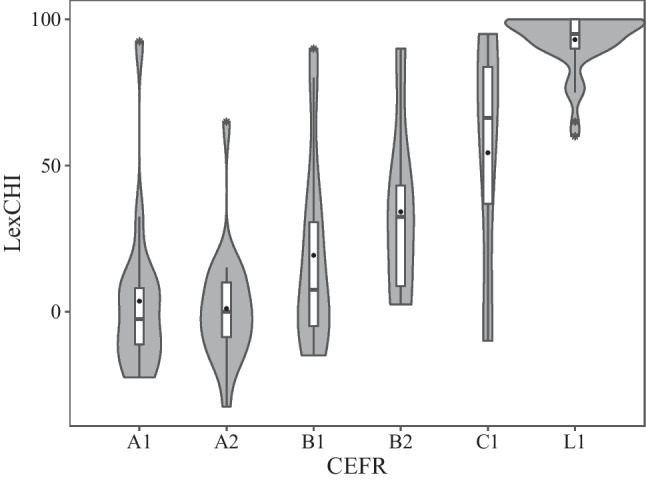
Violin plots showing the distribution of LexCHI scores over self-reported proficiency levels in the Common European Framework of Reference (CEFR). The dots within boxplots indicate the means. The CEFR has three tiers of proficiency levels: A1 (Beginner) & A2 (Elementary) as Basic Users, B1 (Intermediate) & B2 (Upper intermediate) as Independent Users, and C1 (Advanced) & C2 (Proficient) as Proficient Users

**Fig. 5:**
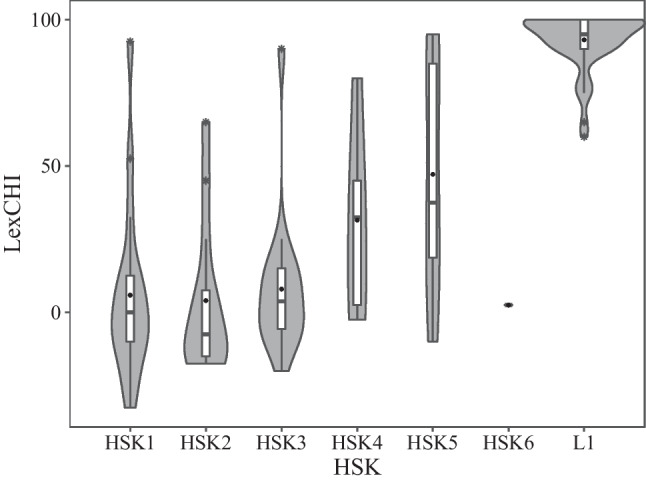
Violin plots showing the distribution of LexCHI scores over proficiency levels in Hanyu Shuiping Kaoshi (HSK). The six levels in HSK correspond to A1, A2, B1, B2, C1 and C2 in the CEFR

## Appendix 1: Stimuli of LexCHI in Experiment 1

The original set of 120 items were used in Experiment 1.

Sixty items of two-character Chinese word (with English translations in brackets):

- 徜徉 (wander), 踉跄 (stagger), 襁褓 (swaddling), 涟漪 (ripple), 踌躇 (hesitate), 惆怅 (melancholy), 璀璨 (luminous), 蹉跎 (wasted), 褴褛 (ragged), 囫囵 (whole), 缥缈 (ethereal), 俸禄 (salary), 狰狞 (ferocious), 匍匐 (creep), 滂沱 (torrential), 蹒跚 (stumble), 鹧鸪 (francolin), 恍惚 (trance), 憧憬 (longing), 朦胧 (hazy), 徘徊 (linger), 傀儡 (puppet), 蹂躏 (ravage), 瑕疵 (defect), 漩涡 (swirl), 鞠躬 (bow), 邋遢 (sloppy), 憔悴 (haggard), 侏儒 (dwarf), 烹饪 (cooking), 瘫痪 (paralysis), 亵渎 (profanity), 咳嗽 (cough), 吩咐 (order), 辉煌 (glorious), 俘虏 (captive), 唠叨 (nag), 脂肪 (fat), 羡慕 (envy), 瘟疫 (plague), 悬崖 (cliff), 颤抖 (tremble), 痊愈 (recover), 栅栏 (fence), 贿赂 (bribe), 贪婪 (greedy), 慷慨 (generous), 寂寞 (lonely), 锻炼 (workout), 谨慎 (cautious), 矛盾 (contradiction), 卑鄙 (despicable), 喉咙 (throat), 祈祷 (prayer), 愤怒 (anger), 骄傲 (proud), 淘汰 (eliminate), 熟悉 (familiar), 掌握 (grasp), 问题 (problem)

Sixty nonword items:

- 缤摹, 鳟踞, 亦筷, 蚯践, 粤抄, 巅妾, 锉洽, 猥砂, 诠疮, 拮娥, 寨禧, 炭鹑, 磷祥, 晴榻, 裳妊, 抒泵, 葵娠, 荟锐, 倚籍, 暮徙, 窍俭, 赠瞻, 霰驻, 鹌棋, 蜥浆, 谜蜍, 耕韵, 逸滞, 狐镀, 咸裕, 馅俐, 呻椒, 罕寝, 炫萃, 鸣蜓, 恒馁, 荧瓷, 契砣, 拱疹, 涵浅, 衔侈, 颁昧, 抹峨, 歪衍, 磁窿, 敞螺, 漆蝠, 濒愧, 嘲塘, 寰港, 晶嫩, 蝉税, 洪噪, 禁蕾, 糖描, 痴策, 辩菜, 舒震, 糊悲, 身候

## Appendix 2: Stimuli in the translation task

Sixty items were presented in the translation task (Task 4) in Experiments 1 & 2.

Thirty English words were used for in the English-to-Chinese translation:blanket, dignity, quilt, enquiry, nerve, curse, palm, pyjamas, bandage, sequence, misery, infection, pest, shelf, torch, missile, mayor, lawn, friction, lightning, stationery, appendix, fountain, tomb, drawer, vowel, foam, exception, beverage, dawn

Thirty Chinese words were used in the Chinese-to-English translation (with English translations in brackets):

- 干旱 (drought), 绝望 (despair), 屠夫 (butcher), 代词 (pronoun), 烟囱 (chimney), 下巴 (chin), 橡皮 (rubber), 沙漠 (desert), 来源 (source), 程度 (extent), 冰山 (iceberg), 背心 (vest), 乞丐 (beggar), 补偿 (compensation), 潮汐 (tide), 拳头 (fist), 难民 (refugee), 燃料 (fuel), 诗人 (poet), 无知 (ignorance), 花费 (expenditure), 商人 (merchant), 物质 (substance), 小丑 (clown), 珍珠 (pearl), 崇拜 (worship), 直觉 (instinct), 虫子 (worm), 裁缝 (tailor), 短缺 (shortage)

## Appendix 3: Items of LexCHI

The 60 items are included in LexCHI.

Forty items of two-character Chinese word (with English translations in brackets):

- 烹饪 (cooking), 喉咙 (throat), 襁褓 (swaddling), 瑕疵 (defect), 咳嗽 (cough), 囫囵 (whole), 问题 (problem), 璀璨 (luminous), 祈祷 (prayer), 瘟疫 (plague), 亵渎 (profanity), 朦胧 (hazy), 愤怒 (anger), 匍匐 (creep), 蹂躏 (ravage), 涟漪 (ripple), 羡慕 (envy), 唠叨 (nag), 滂沱 (torrential), 痊愈 (recover), 骄傲 (proud), 瘫痪 (paralysis), 卑鄙 (despicable),恍惚 (trance), 狰狞 (ferocious), 蹉跎 (wasted), 憧憬 (longing), 淘汰 (eliminate), 贿赂 (bribe), 脂肪 (fat), 吩咐 (order), 掌握 (grasp), 邋遢 (sloppy), 俸禄 (salary), 熟悉 (familiar),寂寞 (lonely), 傀儡 (puppet), 惆怅 (melancholy), 慷慨 (generous), 徘徊 (linger)

Twenty nonword items:

- 鹌棋, 颁昧, 亦筷, 寰港, 裳妊, 呻椒, 糖描, 诠疮, 涵浅, 晶嫩, 抒泵, 缤摹, 锉洽, 抹峨, 咸裕, 晴榻, 契砣, 漆蝠, 巅妾, 磷祥

## Appendix 4: Additional results in Experiment 2

1. Correlations in Experiment 2 (Table 10)

**Table 10:** Correlations of LEXTALE_CH (Task 1) and LexCHI (Task 2) against the cloze test (Task 3), the translation task (Task 4) and the self-ratings of Chinese ability (Experiment 2, *N* = 105)

	Task 1: LEXTALE_CH	Task 2: LexCHI
Task 3: Cloze test	0.78***	0.83***
Task 4: Translation	0.64***	0.70***
Average self-rated Chinese ability	0.76***	0.78***
Self-rated Chinese ability (Listening)	0.66***	0.69***
Self-rated Chinese ability (Speaking)	0.66***	0.68***
Self-rated Chinese ability (Reading)	0.79***	0.81***
Self-rated Chinese ability (Writing)	0.73***	0.75***

* *p* < .05, ** *p* < .01, **** p* < .001

2. Detailed results of the regression analysis in Experiment 2

**Table 11:** Results of the regression analysis (LexCHI scores entered first)

	*R*^2^, adjusted *R*^2^	Estimate (SE)	*t* value	*p*	*η* ^*2*^
Step 1	0.682, 0.6789				
LexCHI		0.56203 (0.03782)	14.86	< .001	0.68
Step 2	0.7048, 0.699				
LexCHI		0.56203 (0.03661)	15.350	< .001	0.70
LEXTALE_CH		0.30580 (0.10888)	2.809	< .01	0.07

Following the suggestion of one reviewer, an additional regression analysis was conducted in which the LEXTALE_CH scores were entered in the first step to predict accuracy of the cloze test. Like the regression analysis reported at the end of Experiment 2 (see Table 11), we first address the issue of the collinearity by orthogonalising the LexCHI scores in a linear model in which the LexCHI scores were predicted by the LEXTALE_CH scores. Then, in the regression analysis, the LEXTALE_CH scores were entered in the first step to predict accuracy of the cloze test, and the LexCHI scores were entered in the second step. As can be seen from Table 12, in the first step, LEXTALE_CH was a significant predictor. In the second step, LEXTALE_CH and LexCHI were both significant predictors. The LexCHI scores uniquely accounted for almost 10% of the variance in the cloze test accuracy as indicated by the increase of *R*^2^ in the second step.

**Table 12:** Results of the regression analysis (LEXTALE_CH scores entered first)

	*R*^2^, adjusted *R*^2^	Estimate (SE)	*t* value	*p*	*η* ^*2*^
Step 1	0.6092, 0.6054				
LEXTALE_CH		0.83662 (0.06603)	12.671	< .001	0.61
Step 2	0.7048, 0.699				
LEXTALE_CH		0.83662 (0.05767)	14.508	< .001	0.67
LexCHI		0.39734 (0.06913)	5.747	< .001	0.24

3. Distributions of LexCHI scores in Experiment 2

In developing the Italian extension of LexTALE, Amenta et al. (2020) asked non-native speakers in the validation study to report their L2 proficiency according the CEFR (Common European Framework of Reference) levels and reported the distributions of LexITA scores over CEFR levels. We also asked the L2 group in Experiment 2 to rate the CEFR proficiency of Chinese reading using the self-assessment grid provided by the CEFR. The distributions of LexCHI scores over self-rated CEFR levels are plotted Fig. 4 in together with the L1 group. In addition, we asked these participants to rate their proficiency of Chinese reading based on the HSK levels. Figure 5 plots the distributions of LexCHI scores over the self-rated HSK levels as well as the L1 group's score.

The question for rating the CEFR levels in terms of Chinese reading was framed as follows: ***Select the description that best summarises your ability to read Chinese:***

- *I can understand familiar names, words and very simple sentences, for example on notices and posters or in catalogues.*
- *I can read very short, simple texts. I can find specific, predictable information in simple everyday material such as advertisements, prospectuses, menus and timetables and I can understand short simple personal letters.*
- *I can understand texts that consist mainly of high-frequency, everyday or job-related language. I can understand the description of events, feelings and wishes in personal letters.*
- *I can read articles and reports concerned with contemporary problems in which the writers adopt particular attitudes or viewpoints. I can understand contemporary literary prose.*
- *I can understand long and complex factual and literary texts, appreciating distinctions of style. I can understand specialised articles and longer technical instructions, even when they do not relate to my field.*
- *I can read with ease virtually all forms of the written language, including abstract, structurally or linguistically complex texts such as manuals, specialised articles and literary works.*

The question for rating the HSK levels of Chinese proficiency was framed as follows: ***Select the description that best summarises your understanding and use of Chinese:***

- *I can understand and use very simple Chinese phrases, meet basic needs for communication and possess the ability to further my Chinese language studies.*
- *I have an excellent grasp of basic Chinese and can communicate in simple and routine tasks requiring a simple and direct exchange of information on familiar and routine matters.*
- *I can communicate in Chinese at a basic level in daily, academic and professional life. I can manage most communication in Chinese when travelling in China.*
- *I can converse in Chinese on a wide range of topics and are able to communicate fluently with native Chinese speakers.*
- *I can read Chinese newspapers and magazines, enjoy Chinese films and plays and give a full-length speech in Chinese.*
- *I can easily comprehend written and spoken information in Chinese and can effectively express myself in Chinese, both orally and on paper.*

## Footnotes

1 When the ratio of words versus nonwords is not 2:1, the normalised Ghent test score can be calculated using this equation: $\frac{{\mathrm N}_\text{yes to words}-\frac{{\mathrm N}_\text{words}}{{\mathrm N}_\text{nonwords}}\times{\mathrm N}_\text{yes to nonwords}}{{\mathrm N}_\text{words}}.$

2 We used demographic filters in Prolific to recruit participants with English (the L2 group) or Chinese (the L1 group) as their first language. To qualify for the L2 group (naive English speakers), participants were expected to obtain a minimal LexTALE score of 80% which is the recommended 80% cut-off score for English speakers with advanced proficiency (Lemhöfer and Broersma, 2012). As mentioned in the Introduction, the LexTALE score was calculated as follows $\frac{\mathrm{number}\;\mathrm{of}\;\mathrm{correct}\;\mathrm{words}+2\ast\mathrm{number}\;\mathrm{of}\;\mathrm{correct}\;\mathrm{nonwords}}{80}$ . Similarly, to qualify for the L1 group, participants should obtain a minimal score of 80% for LEXTALE_CH and for LexCHI and the tests scores were computed as follows: character score = $\frac{\mathrm{number}\;\mathrm{of}\;\mathrm{correct}\;\mathrm{characters}+2\ast\mathrm{number}\;\mathrm{of}\;\mathrm{correct}\;\mathrm{noncharacters}}{120}$, LexCHI score = $\frac{\mathrm{number}\;\mathrm{of}\;\mathrm{correct}\;\mathrm{words}+2\ast\mathrm{number}\;\mathrm{of}\;\mathrm{correct}\;\mathrm{nonwords}}{80}$. Using such criteria, seven participants were swapped across groups in two steps. First, three participants recruited as native Chinese speakers were reassigned to the L2 group because their LexTALE scores were higher than 80% (mean = 96.25%, range = 93.75–100%), whereas their scores of LEXTALE_CH and LexCHI were much lower than 80% (LEXTALE_CH: mean = 54.44%, range = 54.17–63.33%; LexCHI: mean = 57.50%, range = 45–72.5%). Among these three participants, one participant indicated English as first language, and two participants indicated Chinese as first language. Second, four participants recruited as native English speakers were reassigned to the L1 group because their scores of LEXTALE_CH and LexCHI were higher than 80% (LEXTALE_CH: mean = 85.83%, range = 83.3–90.83%; LexCHI: mean = 99.06%, range = 96.25–100%), while their LexTALE scores were lower than 80% (mean = 70.31%, range = 66.25–72.5%). These four participants all indicated Chinese as first language.

3 Their scores of English LexTALE were very high (mean = 96.93%, range = 91.25–100%), while their scores of LEXTALE_CH and those of LexCHI were relatively low (LEXTALE_CH: mean = 55.78%, range = 42.5–84.17%; LexCHI: mean = 51.83%, range = 38.75–97.5%, computed as the equations in Footnote 2).

4 These two participants had low scores for LEXTALE_CH, LexCHI and LexTALE (all < 80%, computed as the equations in Footnote 2).

5 The Cronbach's alpha for was 0.823 for the L1 group and 0.881 for the L2 group. The split-half correlation was 0.728 (Spearman-Brown corrected: 0.842) for the L1 group and 0.764 (Spearman-Brown corrected: 0.866) for the L2 group.

6 The cut-off score is 85 (%) if using the original equation by Lemhöfer and Broersma (2012) instead of the normalised Ghent score. Because the normalised Ghent score is linear equivalent of the score obtained with the original equation, the two cut-off scores have the same values of the AUC, sensitivity and specificity. We also note here that native Chinese speakers typically have a LexCHI score above 90% (with low SD) when using the original equation. Similarly, LexTALE scores of 90% are typical for native English speakers. Therefore, LexTALE and LexCHI seem to have similar difficulty levels.
